# The Changing Relationship Between Hobby Engagement and Substance Use in Young People: Latent Growth Modelling of the Add Health Cohort

**DOI:** 10.1007/s10964-024-02047-x

**Published:** 2024-07-16

**Authors:** Jessica K. Bone, Daisy Fancourt, Jill K. Sonke, Feifei Bu

**Affiliations:** 1 Research Department of Behavioural Science and Health, Institute of Epidemiology & Health Care, University College London, London, UK; 2 Center for Arts in Medicine, University of Florida, Gainesville, FL USA

**Keywords:** Creative, Extracurricular, Hobbies, Alcohol, Tobacco, Marijuana

## Abstract

Cross-sectional and some longitudinal evidence suggests doing hobbies can reduce substance use, but findings have been inconsistent, and whether associations differ across adolescence remains unclear. This study included 7454 Add Health participants (50% female, 77% White, age mean=14.95 and SD = 1.56). Participants were split into three groups, according to whether they were early (aged 11–14 at baseline), mid (aged 15–16), or late (aged 17–20) adolescents at baseline. The trajectories of binge drinking, marijuana, and tobacco use were analysed in latent growth models across Waves 1–5 (1994–2018). Concurrent associations between substance use and hobby engagement were tested at Waves 1–3 separately in the three age groups. Doing hobbies more frequently was associated with lower odds of binge drinking and marijuana and tobacco use in early adolescence. Although there was initially a similar protective association in mid and late adolescence, this had reversed by Wave 3 for binge drinking and marijuana use, when participants were young adults. This change in the association could be a result of differing social contexts, changes in peer influence, or an indication that creative hobbies are particularly beneficial. It could explain previous inconsistent findings and demonstrates the importance of considering developmental differences when investigating engagement in hobbies.

## Introduction

Adolescence has been characterized as a period of increased risk taking (Steinberg, 2004). Substance use is most likely to first occur during adolescence, with frequency of use increasing from mid-adolescence and peaking in early adulthood (Degenhardt et al., 2016). Alcohol, tobacco, and marijuana are used most frequently (Degenhardt et al., 2016). In the US, 16.7% of 12–17-year-olds reported drinking alcohol, 11.5% reported using marijuana, and 4.8% reported using tobacco during 2022 (Centre for Behavioural Health Statistics and Quality, 2023). Whilst some substance use may be normative, preventing adolescents from becoming alienated for peers (Thrash & Warner, 2016), it has also been linked to a range of negative outcomes, including affective and anxiety disorders, self-harm and suicide, cognitive impairment, lower educational attainment, criminalised activity, and poorer health later in life (Hall et al., 2016). Additionally, adolescents may be at higher risk of becoming dependent on substances due to ongoing neurological development (Conrod & Nikolaou, 2016). Identifying ways to reduce problematic youth substance use thus remains a priority. One potential strategy is engaging in hobbies or extracurricular activities. However, evidence on the association between youth hobby engagement and substance use is inconsistent. Additionally, research has not properly accounted for the developmental trajectories of substance use, or potential confounders of the association with hobby engagement, and has measured overall substance use (grouping various substance types together), masking any differences in these associations. This study aims to explore the trajectory of substance use across adolescence and early adulthood, testing the association between hobby engagement frequency and binge drinking, marijuana use, and tobacco use at three separate timepoints in three age groups.

Hobbies are voluntary productive activities, distinguished from other leisure activities because they typically involve skill, identity, and self-development and are not done for money (Daily, 2018). In a review, many cross-sectional studies showed a link between extracurricular activity participation and lower rates of substance use (Feldman & Matjasko, 2005). For example, participating in team sports has been associated with a lower risk of having tried smoking, and participating in other clubs (e.g. Scouts) with a lower risk of having tried alcohol in youth aged 10–14 in the US (Adachi-Mejia et al., 2014). In contrast, in 14-year-olds in South Africa, those who had drunk alcohol, smoked cigarettes, or used marijuana in the last month were more likely to be involved in a range of leisure activities (Tibbits et al., 2009). Yet these cross-sectional studies provide a snapshot of the association without accounting for the developmental trajectories of substance use, and cannot separate differences due to age, period, or cohort effects.

An updated review suggested that extracurricular participation may increase subsequent substance use when examined longitudinally (Feldman Farb & Matjasko, 2012), although this finding was limited to sports participation. More recent longitudinal studies in several countries have found that participating in organised leisure activities can reduce subsequent smoking and drinking, including in Czechia (Badura et al., 2017), Taiwan (Chen et al., 2019), and the US (Eisman et al., 2017). A South African study also found that young people with more positive perceptions of their leisure activities were less likely to have increasing substance use over the subsequent three years, although those who felt more able to plan their leisure activities were more likely to have increasing substance use (Weybright et al., 2016). Focussing more specifically on hobbies that involve creative engagement or expression, some research has found that reading for pleasure (Mak & Fancourt, 2020) and performance and fine arts (Denault et al., 2009) are associated with less subsequent alcohol use and smoking. Yet, this evidence also is not consistent (Barber et al., 2001).

There are several further limitations of existing research. Studies have used indices of ‘delinquent’ (e.g., Rose-Krasnor et al., 2006) or ‘risky’ behaviours (e.g., Zarrett et al., 2009) as outcomes, which are not only problematic terms (Bone et al., 2022), but may have obscured associations by grouping substance use with other behaviours. Different mechanisms are likely to link hobby engagement to distinct outcomes like substance use, criminalised behaviour, and reportedly antisocial behaviour (Fancourt et al., 2021). Previous research has also used binary indicators of activity engagement (Feldman Farb & Matjasko, 2012), without assessing its frequency. Demonstrating a dose-response relationship between engagement frequency and substance use would provide further evidence towards a putative causal relation.

Furthermore, previous evidence on the associations between hobby engagement and substance use could be a result of confounding by a range of factors (Mak & Fancourt, 2020). There is a social gradient in both hobby engagement (Mak & Fancourt, 2021) and substance use (Green et al., 2024), with both types of behaviour influenced strongly by sociodemographic factors. These factors include adolescents’ age, gender, race, education, language spoken at home, parental socioeconomic position (as indicated by factors such as education, marital status, and household income), and neighbourhood characteristics (e.g., urbanicity). Parental substance use is also important for adolescents’ behaviour (Green et al., 2024). These factors must be assessed to test whether associations between hobby engagement and substance use are due to self-selection, or because doing hobbies can lead to reductions in substance use. Research must also account for the fact that adolescents spend much of their time at school, and adolescents within schools are more similar to each other than to adolescents at other schools, due to neighbourhood and school segregation among other factors (Thrash & Warner, 2016).

The relationship between hobby engagement and substance use may also differ according to sociodemographic factors such as gender (Feldman Farb & Matjasko, 2012). On average, females are more likely to engage in hobbies (Mak & Fancourt, 2021) and less likely to use substances (Green et al., 2024; Patrick & Schulenberg, 2013). There is some cross-sectional evidence in 12–17-year-olds in the US that the association between extracurricular activities and substance use is stronger in females than males (Kenney & Dennis, 2019). Yet, in 13–14 year olds in Poland, extracurricular involvement was more strongly associated with lower substance use in males than females (Habib et al., 2014). It therefore remains unclear whether there are robust gender differences in these relationships, which direction they operate in, and whether they occur across all stages of adolescence.

## Current Study

Engaging in hobbies is a potential strategy for reducing youth substance use, but previous evidence is inconsistent. Studies have not accounted for the developmental trajectories of substance use, potential confounders of the association with hobby engagement, or considered the frequency of engagement. This study aimed to investigate whether engagement in hobbies was associated with substance use in adolescence after accounting for a range of confounders. To address the lack of research on specific substances, it measured the most used substances separately (alcohol, marijuana, tobacco). The trajectory of each type of substance use was modelled across adolescence and early adulthood, with the association between hobby engagement frequency and substance use explored at three separate timepoints. Models were estimated separately in three age groups (early, mid, and late adolescents at baseline), to separate potential age and cohort effects. It was hypothesized that more frequent hobby engagement would be associated with lower rates of substance use across all substance types, waves, and age groups. In exploratory sensitivity analyses, this study also aimed to test whether these associations differed according to gender.

## Methods

### Sample

Participants were drawn from the National Longitudinal Study of Adolescent to Adult Health (Add Health), which includes a nationally representative sample of US adolescents who were in grades 7–11 (aged 11–20 years) during the 1994–95 school year. Participants have been followed for five waves over 22 years, from adolescence into adulthood (Harris et al., 2019). The Add Health restricted-use data were used for this study. Participants with complete data on hobby engagement, substance use, and covariates at Waves 1 (1994–1995), 2 (1996), and 3 (2001–2002) of Add Health were eligible for inclusion. Participants had to complete all three waves because at least three repeated measures per individual are required to estimate growth curves. Substance use data from Waves 4 (2008–2009) and 5 (2016–2018) were also analysed, but participants did not have to complete these waves to be eligible. At Wave 1, 18,924 young people participated, of whom 13,568 participated at Wave 2 and 10,828 at Wave 3. Excluding those with missing data on study variables (*n* = 3374) left a final analytical sample of 7454 participants.

### Ethical Approval

All participants gave informed consent, and this analysis has Institutional Review Board approval from the University of Florida (IRB201901792) and ethical approval from University College London Research Ethics Committee (project 18839/001).

### Measures

#### Hobby engagement

At Waves 1–2, Add Health included a single question “During the past week, how many times did you do hobbies, such as collecting baseball cards, playing a musical instrument, reading, or doing arts and crafts?” Response options were not at all, one or two times, three or four times, or five or more times. At Wave 3, the question was repeated, with further examples of hobbies added: “In the past seven days, how many times did you engage in a hobby such as working on a collection, playing cards or board games, arts and crafts, drama, playing a musical instrument or singing with a group, or shopping just for fun?” Response options, ranging from not at all to seven or more times, were categorised as in Waves 1–2.

#### Binge drinking

As low levels of alcohol use are very common, this study focussed specifically on binge drinking, which can have worse health consequences (Kuntsche et al., 2017). One binary indicator of binge drinking was created at each wave. Binge drinking was self-reported at Waves 1–3 as whether participants had 5 or more drinks in a row on any day over the past 12 months (never, one or more days). At Waves 4–5, binge drinking was instead reported as 4 or more drinks in a row for females and 5 or more drinks in a row for males, in line with the CDC definition of binge drinking (Centres for Disease Control and Prevention, 2022).

#### Marijuana use

One binary measure of marijuana use in the last 30 days (yes, no) was created by combining responses to two questions. No marijuana use was indicated by (a) participants reporting that they had never tried marijuana or (b) stating that they had not used it during the past 30 days. Participants were recorded as using marijuana if they reported doing so one or more times in the past 30 days (in Waves 1–3) or on one or more of the past 30 days (Waves 4–5).

#### Tobacco use

One binary measure of tobacco use in the last 30 days (yes, no) was created by combining responses to three questions. No tobacco use was indicated by participants reporting that (a) they had never tried smoking or (b) had not smoked cigarettes on any of the last 30 days and (c) had not used chewing tobacco (such as Redman, Levi Garrett, or Beechnut) or snuff (such as Skoal, Skoal Bandits, or Copenhagen) on any of the last 30 days. Those who had smoked cigarettes or used chewing tobacco or snuff on one or more of the last 30 days were classified as tobacco users.

#### Covariates

Demographic, socioeconomic, and substance use covariates were measured at Wave 1. Demographic factors were age (years), gender (male, female), level of education (school grade), race (White, Black or African American, Asian or Pacific Islander, Other [including Hispanic, American Indian/Native American, Other]), first language spoken at home (English, Other), urbanicity of home area (urban, suburban, rural), and parent marital status (married, never married, widowed, divorced, or separated). Socioeconomic factors were annual household income (thousand US dollars) and parent education (less than high school, high school, some college, college graduate or professional training). Parental substance use factors were whether parents currently smoked (yes, no) and frequency of parent alcohol use (rated 0–5; never to nearly everyday).

### Statistical Analyses

First, characteristics of the sample were examined using descriptive statistics. To allow for differentiation between potential age (time) effects and cohort effects, participants were split into three age groups and analyses performed separately by age group. In line with the stages of adolescence (Salmela-Aro, 2011), and the distribution of age in the sample, early adolescents were 11–14 years (*n* = 2562), mid adolescents 15–16 (*n* = 3176), and late adolescents 17–20 (*n* = 1716) at Wave 1. Group naming related to participants’ age at Wave 1, with follow-up waves one, six, 15, and 22 years after Wave 1 on average. Those who were mid and late adolescents at Wave 1 were over the legal drinking age (21 years) at Wave 3, with the late adolescent group having moved into early adulthood.

**Fig. 1:**
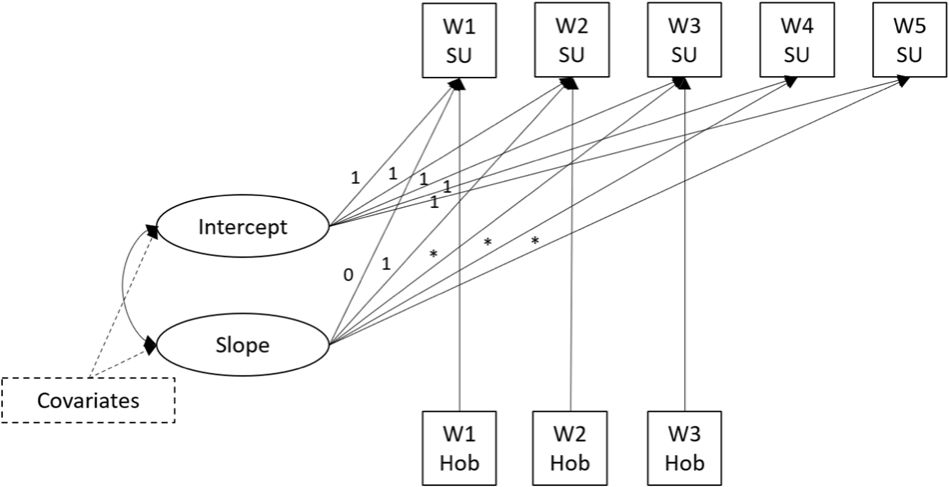
Final latent growth model. SU: substance use (modelled separately for each substance type; binge drinking, marijuana use, tobacco use). Hob: frequency of hobby engagement. Covariates were age, gender, education, race/ethnicity, first language, urbanicity, household income, parent marital status, parent education, parent smoking, parent alcohol use. For binge drinking and marijuana use, the associations between hobby engagement and substance use were allowed to differ across waves. For tobacco use, this association was held constant at Waves 2 and 3

Data were analysed using latent growth modelling (Preacher et al., 2008), including each type of substance use (binge drinking, marijuana, tobacco use) in a separate model. Models aimed to explore how each individual’s hobby engagement related to their substance use at different times and ages, given the developmental trajectory of substance use. They therefore estimated the trajectory of substance use from Waves 1–5, with hobby engagement at Waves 1–3 included as a time-varying variable (Fig. 1). The concurrent associations between hobby engagement and substance use at Waves 1–3 were estimated. All covariates measured at Wave 1 were included as predictors of the model growth factors (intercept and slope).

A series of models were compared in the whole sample for each outcome separately, starting with the simplest model and adding increasing complexity (Table S3). Model 1 included a linear trajectory of substance use, with the slope for each outcome corresponding to the number of years since baseline (0, 1, 6, 13, 21). It also restricted the association between hobby engagement and substance to be constant across Waves 1–3. Model 2 kept the fixed associations between hobbies and substance use, but the shape of the trajectory of substance use was estimated freely with free time score models. The first two time points were set to 0 and 1 for identification purposes. Model 3 included a linear trajectory of substance use (as in Model 1) but allowed the association between hobbies and substance use to differ across waves. Models 4 and 5 estimated the trajectory of substance use freely (as in Model 2) and held the associations between hobbies and substance use constant across two of the three waves (Model 4 = Waves 1–2 fixed; Model 5 = Waves 2–3 fixed). Finally, Model 6 was the most complex, with the trajectory of substance use estimated freely (as in Model 2) and the association between hobbies and substance use allowed to differ across all waves (as in Model 3).

The winning model was selected based on a range of fit statistics (AIC, BIC, sample-size adjusted BIC) as well as chi-square difference tests (Tables S4–S5). For binge drinking, Model 6 was the winning model, which included a freely estimated non-linear trajectory of binge drinking and estimated the association between hobby engagement and binge drinking separately at each wave. In real terms, this meant that the odds of binge drinking did not increase linearly for each one-year increase in age over the 22-year period, and the associations between hobby engagement and binge drinking differed between baseline and follow-ups one and six years later. The same model was selected for marijuana use. The winning tobacco use model was similar with one additional restriction, that the association between hobby engagement and substance use was fixed (did not differ) across Waves 2 and 3 (Model 5; Tables S3–S5).

All models were fitted using full information maximum likelihood estimation with robust standard errors. Models were weighted with the Add Health Waves 1–3 longitudinal weight (Chen & Mullan Harris, 2020) and accounted for sample clustering and stratification. Main analyses, including latent growth modelling, were conducted in Mplus 8 (Muthén & Muthén, 2017), with descriptive statistics produced using Stata 18 (StataCorp, 2023). Mplus syntax for the final models is available in the Supporting Information.

#### Sensitivity analyses

Exploratory sensitivity analyses first tested whether there were gender differences in the associations between hobby engagement and substance use. This was done by refitting the final models for each type of substance use separately according to gender (male, female).

Second, sensitivity analyses explored whether including only participants with complete data on all study variables at Waves 1–3 influenced findings. This involved re-running analyses using different sample eligibility criteria and assessing whether findings were replicated across approaches. Due to issues with estimation and convergence with high proportions of missing data, this was done for just one model (binge drinking in early adolescents). First, the sample included all participants with data available at one or more waves (total *n* = 18,924). Second, the sample included participants who completed Waves 1–3, with missing data on study variables allowed (total *n* = 10,828). Finally, taking a consistent approach to missingness across waves, the sample included participants who completed Waves 1–5, with missing data on study variables allowed (total *n* = 7294). All models were fitted using full information maximum likelihood estimation with robust standard errors.

## Results

After weighting, participants had a mean age of 14.95 (standard deviation [SD] = 1.56), which ranged from 11 to 20, at Wave 1. Overall, 50% were female, 77% were of White race, and 14% were of Black/African American race (Table 1). Early adolescents (*n* = 2562) had a mean age of 13.39 (SD = 0.65), mid adolescents (*n* = 3176) had a mean age of 15.48 (SD = 0.50), and late adolescents (*n* = 1,716) had a mean age of 17.30 (SD = 0.54; Table 1).

**Table 1:** Sample characteristics at Wave 1

	Overall	Early	Mid	Late
	Mean (SD)			
Age (Wave 1)	14.95 (1.56)	13.39 (0.65)	15.48 (0.50)	17.30 (0.54)
Age (Wave 2)	15.82 (1.58)	14.27 (0.71)	16.35 (0.61)	18.14 (0.66)
Age (Wave 3)	21.27 (1.58)	19.74 (0.74)	21.81 (0.69)	23.55 (0.69)
Level of education (school grade)	9.00 (1.46)	7.61 (0.59)	9.52 (0.84)	10.97 (0.73)
Household income (thousand USD)	46.54 (43.15)	44.76 (32.00)	47.89 (49.46)	47.67 (54.19)
Parent alcohol use	1.04 (1.16)	1.08 (1.05)	1.04 (1.22)	0.97 (1.28)
	**Proportion**			
Gender				
Male	50%	49%	49%	55%
Female	50%	51%	51%	45%
Race				
White	77%	78%	77%	74%
Black/African American	14%	13%	14%	17%
Asian or Pacific Islander	3%	3%	2%	3%
Other	6%	6%	7%	6%
First language				
English	95%	96%	95%	92%
Other	5%	4%	5%	8%
Urbanicity				
Urban	31%	31%	31%	32%
Suburban	40%	40%	40%	39%
Rural	29%	29%	29%	29%
Parent marital status				
Married	74%	75%	74%	74%
Never married	5%	5%	5%	4%
Widowed/divorced/separated	21%	20%	21%	22%
Parent education				
Less than high school	9%	8%	10%	10%
High school	32%	33%	30%	32%
Some college	22%	21%	23%	22%
College graduate	37%	38%	37%	36%
Parent currently smoked	30%	32%	30%	27%

Overall *n* = 7454. Early adolescents *n* = 2562. Mid adolescents *n* = 3176. Late adolescents *n* = 1716. Descriptive statistics account for survey design characteristics and weighted. Other race includes Hispanic, American Indian/Native, and Other. All characteristics measured at Wave 1 except age

At Wave 1, 19% of participants had not engaged in hobbies in the past week, 32% engaged 1–2 times, 23% engaged 3–4 times, and 26% had engaged in hobbies 5+ times in the past week. Early adolescents engaged in hobbies more frequently than mid and late adolescents, but these proportions remained relatively stable over time (Table S1).

### Binge Drinking

**Fig. 2:**
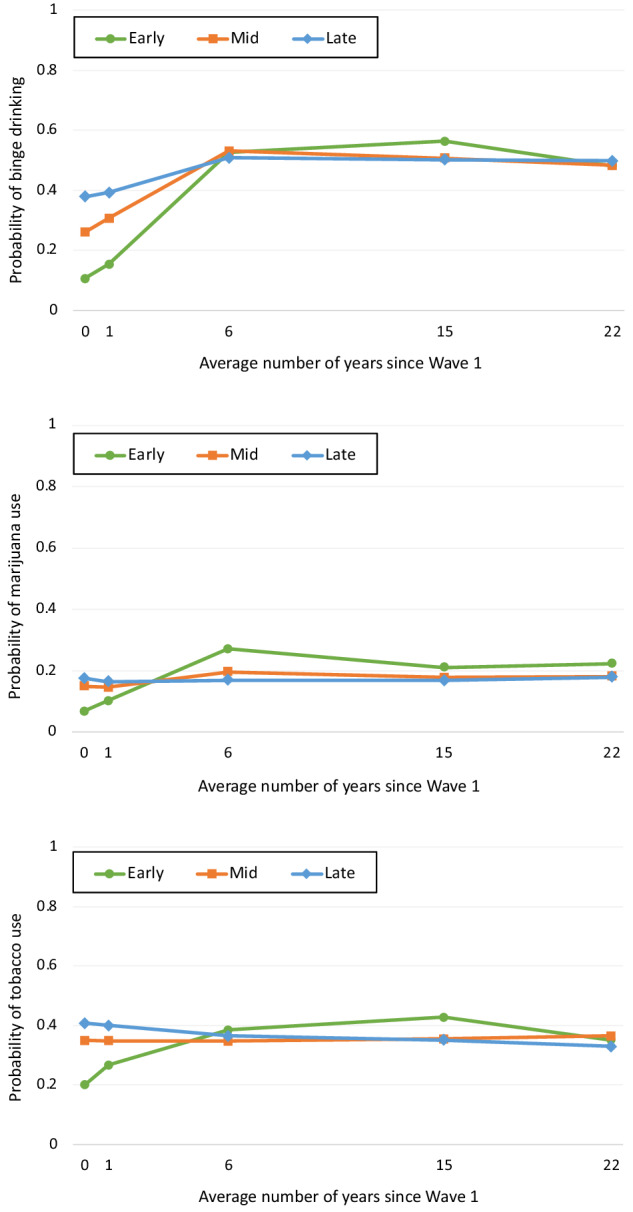
Estimated probabilities of substance use from latent growth models excluding hobby engagement. Models conditional on the mean values of all covariates, estimated separately by age group. Early adolescents were 11–14 years, mid adolescents were 15–16, and late adolescents were 17–20 at Wave 1

At Wave 1, 11% of early adolescents, 28% of mid adolescents, and 39% of late adolescents reported binge drinking (5 or more drinks in a row) on any day over the past 12 months (Table S2). This increased over time, peaking at 57% in Wave 4 in early adolescents and 54% in Wave 3 in both older groups. The growth trajectory of binge drinking showed a non-linear pattern conditional on the mean value of covariates, with the largest increases from Wave 1 to 3, which then plateaued to Wave 5 (Fig. 2). Early adolescents had the steepest slopes from Wave 1 to 3, showing the greatest increase in probability of substance use. The probability of binge drinking did also increase gradually during this period in mid and late adolescents’ transition to adulthood, although it started from a higher point.

**Fig. 3:**
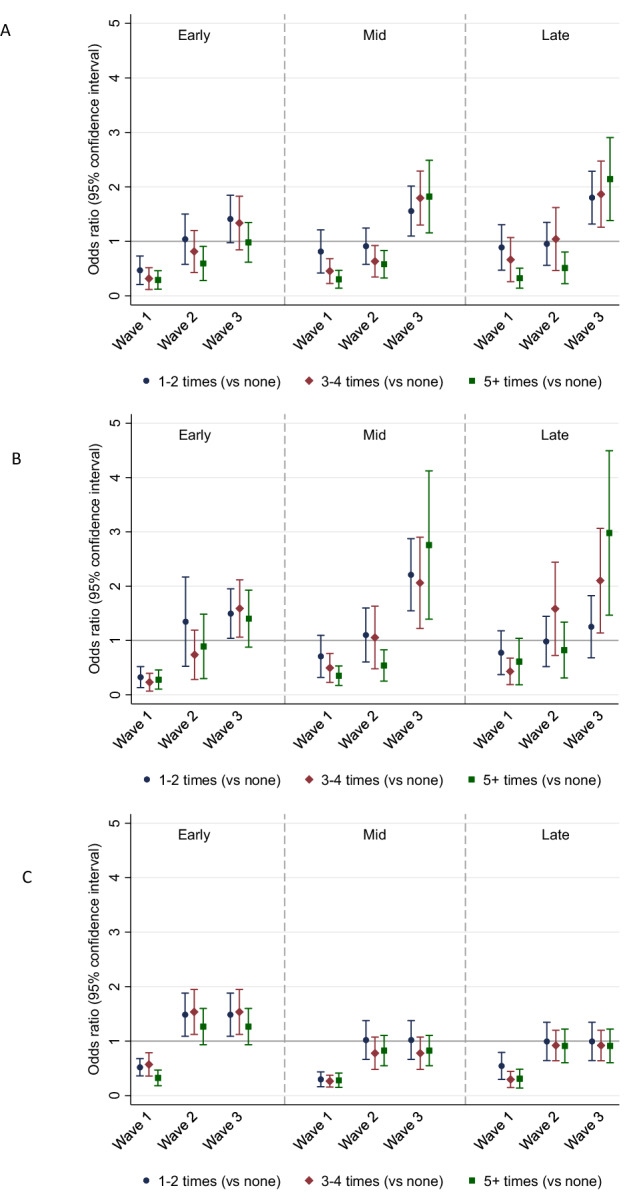
Concurrent associations between hobby engagement frequency in the last week and binge drinking (**A**), marijuana use (**B**), and tobacco use (**C**) at each wave from the latent growth models. Engagement frequency was none (reference category), 1–2 times, 3–4 times, or 5+ times. Models estimated separately by age group and adjusted for all covariates. Early adolescents were 11–14 years, mid adolescents were 15–16, and late adolescents were 17–20 at Wave 1

For early adolescents, there was a dose-response relationship between more frequent hobby engagement and lower odds of binge drinking, which was strongest at Wave 1 (Fig. 3, Table S6). At Wave 2 (one year later), only the most frequent engagement was associated with lower odds of binge drinking. At Wave 3 (six years after Wave 1), there was no evidence for associations between engagement and binge drinking in early adolescents. In mid and late adolescents, there was evidence that the most frequent levels of hobby engagement were associated with lower odds of binge drinking at Waves 1 and 2. However, these relationships were reversed in both older groups at Wave 3, with engagement at any frequency associated with higher odds of binge drinking than not engaging in hobbies.

### Marijuana Use

At Wave 1, 7% of early, 15% of mid, and 18% of late adolescents reporting using marijuana one or more times in past 30 days. This also increased over time, with the highest marijuana use at Wave 3 (early 28%, mid 23%, late 21%). The growth trajectory of marijuana use was also non-linear conditional on the mean value of covariates, increasing from Wave 1 to Wave 3 and then decreasing gradually to Wave 5 in early adolescents, but remaining relatively stable in mid and late adolescents.

In early adolescents, there was again a strong association between hobby engagement and lower odds of marijuana use at Wave 1. However, there was no evidence for an association at Wave 2 and, at Wave 3, the association was reversed, meaning that hobby engagement was associated with higher odds of marijuana use for early adolescents. This pattern was similar for mid and late adolescents (who were early adults by Wave 3). However, the protective association between engagement and marijuana use at Wave 1 was smaller with increasing age. There was also some evidence that the most frequent hobby engagement (5+ times) was associated with lower odds of marijuana use at Wave 2 in mid adolescents. Yet, in both older groups, engagement was associated with higher odds of marijuana use at Wave 3, with larger ORs than in early adolescents.

### Tobacco Use

At Wave 1, 19% of early, 31% of mid, and 38% of late adolescents reported using tobacco in the last 30 days. This also increased over time, peaking at 43% and 40% in Wave 4 for early and mid adolescents respectively, and 44% in Wave 2 for late adolescents. As with other substances, the trajectory of tobacco use was non-linear, increasing gradually from Wave 1 to 4, followed by a small reduction at Wave 5 in early adolescents. In contrast, in mid and late adolescents, the trajectory decreased gradually from Wave 1 onwards into early adulthood. Unlike other substances, model fitting indicated that the association between hobby engagement and tobacco use should be fixed across Waves 2–3, with a free association at Wave 1 (Tables S3–5).

In early adolescents, there was a protective association between hobby engagement and lower odds of tobacco use at Wave 1. In contrast, at Waves 2 and 3, there was evidence that engaging 1–2 or 3–4 times a week (but not 5+ times) was associated with higher odds of tobacco use in early adolescents. In mid and late adolescents, there was again a protective association between engagement and lower odds of tobacco use at Wave 1. Yet, at Waves 2 and 3, there was no evidence for an association with tobacco use in either older group.

### Sensitivity Analyses

In exploratory analyses, there was very little evidence for gender differences in the associations between hobby engagement and substance use. There were a few exceptions, but these should be treated with caution as subgroups were small (with the sample split by both age and gender), and results were generally similar across genders (Table S7). For example, the association between hobby engagement and binge drinking was stronger in males in the mid and late adolescent groups at Wave 3. There was a gender difference for marijuana use in mid adolescents at Wave 2, with hobby engagement associated with higher odds of marijuana use in males but lower odds in females. However, this finding was not replicated at other waves or in other age groups. By Wave 3, hobby engagement was associated with higher odds of marijuana use in both male and female mid adolescents. Finally, the association between hobby engagement and tobacco use was stronger in females than males in early and mid adolescents, but in opposite directions. In early adolescent females, more hobby engagement was associated with higher odds of tobacco use but, in mid adolescent females, it was associated with lower odds.

In further sensitivity analyses, altering the sample eligibility criteria and approach to missing data did not substantially influence the study findings (Table S8).

## Discussion

Engaging in hobbies is a potential strategy for reducing youth substance use, but previous evidence is inconsistent. Studies have not accounted for the developmental trajectories of substance use, potential confounders of the association with hobby engagement, or considered the frequency of engagement. This study aimed to investigate whether frequency of engagement in hobbies was associated with binge drinking, marijuana, and tobacco use separately, after accounting for a range of confounders. It modelled the trajectory of each type of substance use across adolescence and early adulthood, testing associations with hobby engagement at three timepoints, in three separate age groups. Overall, results indicated a potential initial beneficial association between engaging in hobbies, binge drinking, and marijuana use in adolescence, which then became detrimental in later adolescence and early adulthood.

Considering the trajectories of substance use, early adolescents (aged 11–14 at baseline) had the greatest increases in all types of substance use across the 22-year follow-up. This was particularly evident for binge drinking, the most common form of substance use in all age groups. There were also smaller increases in binge drinking from Wave 1 to 3 (1994–2002) for mid and late adolescents (15–16 and 17–20 at baseline respectively), but binge drinking then stabilised across Waves 4 and 5 (2008–2018). This is consistent with findings from Monitoring the Future, a long-term survey of substance use in the US, which generally finds higher rates of binge drinking in older adolescents, and showed small increases in binge drinking in the 1990s, followed by declines in the 2000s (Johnston et al., 2019). Increases in alcohol use with age are to some extent normative, reflecting increasing exploration and experimentation, and could be considered developmentally appropriate, as it enables youth to adhere to peer norms (Thrash & Warner, 2016). However, binge drinking, involving heavy alcohol consumption, remains a concern for public health. Also consistent with Monitoring the Future findings (Johnston et al., 2019), levels of marijuana use did not change substantially over time in this study. There were slight decreases in tobacco use across the whole study in the two older groups, and from Wave 4 to 5 in the youngest group. It is important to note that the included tobacco use questions did not measure vaping, which has dramatically increased in American youth over the last decade (Johnston et al., 2019). The slow decline in tobacco use in the final three waves of this study thus relate only to cigarette smoking and chewing tobacco. It remains to be explored whether hobby engagement is differentially associated with vaping.

Hobby engagement was most strongly associated with lower odds of binge drinking and marijuana use in early adolescents. Although there was also a protective association of engagement with these substances in mid and late adolescents at Wave 1, this had reversed by Wave 3, with engagement associated with higher odds of binge drinking and marijuana use in young adults. The early positive association between hobby engagement and substance use is in line with evidence from arts-based interventions (Maina et al., 2022), creative activity interventions (Bungay & Vella-Burrows, 2013), and some findings in longitudinal studies of organised leisure time activities (Badura et al., 2017), sports participation (Chen et al., 2019), performance and fine arts (Denault et al., 2009), reading for pleasure (Mak & Fancourt, 2020), and perceptions of leisure activities (Weybright et al., 2016).

Although this study focussed on concurrent associations, so could not test the direction of the relationship between engagement and substance use, causal effects are mechanistically plausible. Hobbies could lower substance use by reducing cravings (Silverman, 2019) and boredom, which is associated with higher rates of substance use (Wegner & Flisher, 2009). The association between hobbies and lower binge drinking and marijuana use is particularly promising in early adolescents, who showed the greatest increase in substance use with time. Early adolescence may be a sensitive period in which substance use is particularly susceptible to social influences (Blakemore & Mills, 2014). Encouraging the development of and engagement in hobbies could be a promising behavioural approach early in adolescence.

However, this relationship may be reversed for tobacco use, with hobby engagement more protective for mid to late adolescents and young adults. The protective dose-response relationship with hobby engagement was strongest in mid and late adolescents at Wave 1. Additionally, engagement was only associated with higher odds of tobacco use in early adolescents at Waves 2 and 3. This is surprising given that substance use behaviours often cluster, although could be explained by the lower minimum purchasing age for tobacco than alcohol (Apollonio & Glantz, 2016). Tobacco may have been more available to early adolescents than other substances, whereas mid and late adolescents and young adults could more easily have purchased alcohol and marijuana. Further research is needed to identify why the associations between hobby engagement and substance use might differ according to substance type, including whether the availability of substances is a moderator of the association with hobbies.

The change in direction of the association between substance use and hobby engagement later in adolescence and into early adulthood was unexpected but could explain previous inconsistent longitudinal findings (Feldman Farb & Matjasko, 2012). This study aimed to explore whether differences were due to age, period, or cohort effects by splitting the sample into age groups (birth cohorts). Age effects are caused by the developmental processes of aging, whereas period effects result from factors that affect all ages at a particular point in time, and cohort effects are due to birth cohorts having different experiences across time (Keyes & Li, 2011). To some extent, it appeared that associations changed with age. Binge drinking and marijuana use both peaked at mid to late adolescence, consistent with previous findings (Degenhardt et al., 2016). By this point, adolescents are of legal drinking age and likely to have left home and/or gone to college, a well-established risk factor for substance use (Carter et al., 2010). This changing context could alter the association with hobby engagement. Earlier in adolescence, hobbies may be more likely to be structured, supervised, and done in a formal setting (e.g., a school orchestra). Later, hobbies may take place in an informal setting without supervision, where substance use is more likely (e.g., a band with friends). The changing relationship could also be a result of changes in peer influence with age. Adolescents become particularly susceptible to peer influence (Tomova et al., 2021) and peer substance use affects individual alcohol use (Fujimoto & Valente, 2013) and smoking (Fujimoto et al., 2012). Any impacts of hobbies may depend on other participants. If the group encourages substance use, then participation may be detrimental (Mahoney, 2014). Earlier in adolescence, hobbies might provide a positive peer group less likely to use substances. With age, peers become more likely to use substances, and peer influence may increase substance use. Future research should explore the role of peer influence and whether associations differ according to the specific hobbies engaged in.

There was also some evidence of cohort effects. Despite being similar ages (14–15 years), the associations for early adolescents at Wave 2 were different to mid adolescents at Wave 1. This could be due to policy changes between when each birth cohort reaches a certain age. For example, states across the US were decriminalising marijuana and legalising medicinal cannabis throughout the 1990s and early 2000s. It is also possible that differences were due to period effects, as there were protective associations between hobby engagement and substance use in all groups at Wave 1. According to Monitoring the Future, both binge drinking and marijuana use peaked in the late 1990s (Johnston et al., 2019), and then declined into the 2000s. However, it is unclear why this might mean hobby engagement was more beneficial in 1994–1995 than 1996. Changes in measurement can also result in apparent period effects. At Waves 1–2, examples of hobbies included playing a musical instrument, reading, doing arts and crafts, or collecting baseball cards. Music, reading, and arts can be classed as “creative” hobbies in that they involve ingredients including imagination, multi-modal sensory stimulation, and aesthetic engagement (Warran et al., 2022). Arts-based programmes have been developed and found to reduce substance use among young people, including drama, music, photography, and dance interventions (Maina et al., 2022). The creative elements of these activities can provide a way of empowering young people with knowledge about substance use (Maina et al., 2022), as well as enhancing self-esteem, social competence (Bungay & Vella-Burrows, 2013), motivation (Hohmann et al., 2017), goal setting, and problem-solving skills (Daykin et al., 2008).

At Wave 3, Add Health provided broader examples of hobbies in its question, adding working on a collection, playing cards or board games, singing with a group, or shopping just for fun. While group singing (and arguably working on a collection) are still creative, playing games and shopping do not involve the same aesthetic ingredients. Playing cards or board games could be carried out in the context of drinking or gambling (which is itself associated with substance use). Shopping can become problematic in excess, as well as potentially exposing people to substances such as tobacco in shopping vicinities and activating further potentially adverse psychological mechanisms related to consumerism and procrastination (Yip et al., 2014).

Even in the absence of a change in the prompts from Add Health, adolescents’ own definitions of their hobbies may also have changed over time. Late adolescents may be more likely to include live music events or festivals as hobbies, which are associated with increased substance use (Lim et al., 2010). It therefore remains unclear whether it is changes to the measure that explains the inconsistent findings in Wave 3 versus Waves 1–2 for binge drinking and marijuana use, or whether the protective association really does reverse over time. Despite this, these analyses are informative about hobby engagement as defined by young people themselves. They demonstrate that *perceived* hobby engagement has differential associations with substance use. Future research is recommended to further explore whether particular hobbies are uniquely beneficial. Add Health did not include prompts on sports or physical activity, which could activate unique mechanisms to reduce substance use (e.g. affecting biological cravings or enhancing behavioural motivation not to use substances because of their effects on physical performance).

This study has several strengths. Add Health is a large nationally representative study with rich data throughout and beyond adolescence (Harris et al., 2019). Repeated measures in a cohort with a relatively wide age range allowed the examination of the trajectories of substance use, as well as how associations between engagement and substance use differed according to time and age. However, this study also has limitations. The trajectory of hobby engagement could not be modelled as the measure changed over time and was only included in three waves. This meant the association between hobby engagement and substance use could only be examined concurrently, so the direction of the relationship remains unclear. The changing measure might also have influenced the relationship between hobbies and substance use. Future research should test this relationship prospectively to assess whether it could be causal. To facilitate this, longitudinal studies should use consistent measures of hobby engagement across waves. Engagement was only measured with one item, which provided examples of potential hobbies. Yet, the latent growth models enabled the testing of repeated concurrent associations with substance use. This study only included participants who completed Waves 1–3 of Add Health, reducing the sample size. However, longitudinal weights ensured this sample remained representative, and sensitivity analyses indicated that using alternative approaches to missing data did not influence study findings.

Although models were adjusted for a range of demographic, socioeconomic, and parent substance use covariates, unmeasured confounding is still possible. Modelling the trajectories of substance use assumes that there is no time-varying confounding. Add Health did not repeatedly measure hobby engagement in school (e.g., extracurricular activities). This should be explored in future research, as there may be a larger social gradient in out of school activities (Mak & Fancourt, 2021), resulting in additional unmeasured confounding. The measures of gender (male, female; due to availability in Add Health) and race (White, Black, Asian/Pacific Islander, Other; due to small numbers in non-White groups) were overly simplistic. This approach conflates experiences across diverse gender, racial, and ethnic groups, which might be particularly problematic as these groups may not have equal access to hobbies (Bone et al., 2021). Future research should use more diverse samples and collect more nuanced data on race and ethnicity, while considering the persistence of structural racism in communities, schools, and legal systems (Williams, 2012). Although sensitivity analyses tested whether associations between hobby engagement and substance use differed according to gender, findings were not consistent, indicating that these exploratory analyses may have been underpowered. This study measured substance use in the past month (marijuana, tobacco) and year (binge drinking), focussing on recent behaviour instead of the age of substance use onset. Finally, despite the longitudinal design and statistical adjustments for confounders, results remain observational and reverse causation cannot be ruled out, especially since substance use has been found to predict subsequent trajectories of activity participation in adolescence (Eisman et al., 2016).

## Conclusion

Although engaging in hobbies presents a potential strategy for reducing substance use, previous evidence has been inconsistent, and studies have not accounted for developmental changes, potential confounders, or the importance of engagement frequency and separating different types of substance use. This study aimed to investigate whether frequency of engagement in hobbies was associated with binge drinking, marijuana, and tobacco use separately, after accounting for a range of confounders. Modelling the trajectory of each type of substance use across adolescence and early adulthood, associations with hobby engagement were tested at three timepoints in three separate age groups. There was evidence that doing hobbies more frequently was associated with lower odds of binge drinking and marijuana and tobacco use early in adolescence. However, in early adulthood, associations were reversed, as those who engaged in hobbies had higher odds of binge drinking and marijuana use. This change in the association could be a result of differing social contexts, changes in peer influence, or an indication that creative hobbies are particularly beneficial. Although it remains unclear whether hobby engagement causally influences substance use, these findings demonstrate the importance of considering developmental differences when doing research on hobby engagement. For example, interventions promoting engagement in hobbies should take steps to ensure that this does not lead to increases in substance use in late adolescence and early adulthood.

## Supplementary Information

Supplementary Materials
