# Supplementary material for: The Changing Relationship Between Hobby Engagement and Substance Use in Young People: Latent Growth Modelling of the Add Health Cohort
Source: J Youth Adolesc. 2024 Jul 16;54(1):133–45. doi: 10.1007/s10964-024-02047-x (PMC11742730; doi:10.1007/s10964-024-02047-x)
Supplement: Supplementary file 1 — Supplementary Materials [file 10964_2024_2047_MOESM1_ESM.pdf]

Supplementary Materials

Table S1. Proportion of sample reporting hobby engagement at each wave.

Hobby engagement in the past week	Wave 1	Wave 2	Wave 3
	Proportion		
Overall			
None	19%	15%	22%
1-2 times	32%	34%	29%
3-4 times	23%	24%	26%
5+ times	26%	27%	23%
Early adolescents			
None	16%	12%	21%
1-2 times	32%	31%	28%
3-4 times	24%	27%	25%
5+ times	28%	30%	26%
Mid adolescents			
None	20%	16%	21%
1-2 times	33%	35%	30%
3-4 times	22%	23%	27%
5+ times	25%	26%	22%
Late adolescents			
None	24%	21%	24%
1-2 times	33%	38%	28%
3-4 times	21%	21%	28%
5+ times	22%	20%	20%

Note. N=7,454. Descriptive statistics account for survey design characteristics and weighted.

Table S2. Proportion of sample reporting substance use at each wave.

Age group	Wave 1	Wave 2	Wave 3	Wave 4	Wave 5
	Proportion				
Binge drinking					
Overall	23%	28%	54%	53%	47%
Early	11%	18%	53%	57%	49%
Mid	28%	32%	54%	51%	46%
Late	39%	42%	54%	48%	45%
Marijuana use					
Overall	12%	15%	25%	18%	19%
Early	7%	13%	28%	20%	22%
Mid	15%	17%	23%	16%	18%
Late	18%	18%	21%	16%	17%
Tobacco use					
Overall	27%	37%	38%	41%	31%
Early	19%	31%	39%	43%	33%
Mid	31%	39%	38%	40%	30%
Late	38%	44%	37%	39%	30%

Note. N=7,454. Descriptive statistics account for survey design characteristics and weighted. Participants with complete data varied at Wave 4 (overall drinking n=6,561; marijuana n=6,578; tobacco n=6,583) and Wave 5 (overall drinking n=5,265; marijuana n=5,270; tobacco n=5,269).

Table S3. Overview of linear growth models estimated.

Model	Substance use slope	Hobby and substance use relationship	No. parameters
1	W1-5 linear	W1-3 fixed	42
2	W1-2 linear W3-5 free	W1-3 fixed	45
3	W1-5 linear	W1-3 free	48
4	W1-2 linear W3-5 free	W1-2 fixed W3 free	48
**5**	**W1-2 linear W3-5 free**	**W1 free W2-3 fixed**	48
**6**	**W1-2 linear W3-5 free**	**W1-3 free**	51

Note. Bold text indicates winning models.

Table S4. Model fit statistics for all linear growth models estimated.

Model	AIC	BIC	Sample-size adjusted BIC
**Binge drinking**			
1	40,212	40,502	40,369
2	37,718	38,029	37,886
3	38,593	38,925	38,772
4	37,657	37,989	37,836
5	37,680	38,012	37,859
**6**	**37,628**	**37,981**	**37,819**
**Marijuana use**			
1	28,180	28,470	28,337
2	27,354	27,666	27,523
3	27,460	27,792	27,639
4	27,304	27,636	27,483
5	27,275	27,607	27,454
**6**	**27,223**	**27,575**	**27,413**
**Tobacco use**			
1	35,920	36,211	36,077
2	35,512	35,823	35,680
3	35,345	35,677	35,525
4	35,507	35,839	35,686
**5**	35,198	**35,530**	**35,377**
6	**35,186**	35,539	**35,377**

Note. Bold text indicates lowest values and winning models for each outcome.

Table S5. Chi-square difference tests comparing model fit for the whole sample.

Model comparison	Binge drinking	Marijuana use	Tobacco use
2 vs 1	$\chi^2(3) = 1208$, $p < .001$	$\chi^2(3) = 381$, $p < .001$	$\chi^2(3) = 107$, $p < .001$
3 vs 1	$\chi^2(6) = 939$, $p < .001$	$\chi^2(6) = 440$, $p < .001$	$\chi^2(6) = 366$, $p < .001$
4 vs 2	$\chi^2(3) = 38$, $p < .001$	$\chi^2(3) = 38$, $p < .001$	$\chi^2(3) = 3.26$, $p = .353$
5 vs 2	$\chi^2(3) = 33$, $p < .001$	$\chi^2(3) = 58$, $p < .001$	**$\chi^2(3) = 291$, $p < .001$**
6 vs 2	$\chi^2(6) = 64$, $p < .001$	$\chi^2(6) = 78$, $p < .001$	$\chi^2(6) = 166$, $p < .001$
6 vs 3	$\chi^2(3) = 541$, $p < .001$	$\chi^2(3) = 95$, $p < .001$	$\chi^2(3) = 35$, $p < .001$
6 vs 4	$\chi^2(3) = 25$, $p < .001$	$\chi^2(3) = 39$, $p < .001$	$\chi^2(3) = 435$, $p < .001$
6 vs 5	**$\chi^2(3) = 31$, $p < .001$**	**$\chi^2(3) = 26$, $p < .001$**	**$\chi^2(3) = 5.90$, $p = .116$**

Note. Bold text indicates winning model.

Table S6. Concurrent associations between hobby engagement frequency in the last week (none, 1-2 times, 3-4 times, 5+ times) and substance use at each wave from the latent growth models, estimated separately by age group and adjusted for all covariates.

	**Early adolescents** (n=2,562)	**Mid adolescents** (n=3,176)	**Late adolescents** (n=1,716)
	Odds ratio (95% confidence interval)		
**Binge drinking**			
Wave 1			
1-2 times	**0.47 (0.21, 0.73)**	0.81 (0.42, 1.21)	0.89 (0.47, 1.31)
3-4 times	**0.32 (0.12, 0.52)**	**0.45 (0.23, 0.68)**	0.66 (0.26, 1.07)
5+ times	**0.29 (0.12, 0.46)**	**0.30 (0.14, 0.47)**	**0.32 (0.14, 0.51)**
Wave 2			
1-2 times	1.04 (0.58, 1.50)	0.91 (0.58, 1.25)	0.95 (0.56, 1.35)
3-4 times	0.81 (0.43, 1.20)	**0.63 (0.35, 0.92)**	1.04 (0.46, 1.62)
5+ times	**0.60 (0.28, 0.91)**	**0.58 (0.33, 0.83)**	**0.51 (0.22, 0.80)**
Wave 3			
1-2 times	1.41 (0.97, 1.85)	**1.56 (1.10, 2.01)**	**1.80 (1.32, 2.29)**
3-4 times	1.34 (0.84, 1.83)	**1.79 (1.30, 2.29)**	**1.87 (1.26, 2.47)**
5+ times	0.98 (0.62, 1.35)	**1.82 (1.16, 2.49)**	**2.14 (1.38, 2.91)**
**Marijuana use**			
Wave 1			
1-2 times	**0.33 (0.13, 0.52)**	0.71 (0.32, 1.10)	0.78 (0.37, 1.18)
3-4 times	**0.23 (0.07, 0.39)**	**0.50 (0.23, 0.76)**	**0.43 (0.19, 0.68)**
5+ times	**0.28 (0.10, 0.46)**	**0.35 (0.17, 0.53)**	0.61 (0.18, 1.04)
Wave 2			
1-2 times	1.35 (0.52, 2.17)	1.10 (0.61, 1.60)	0.98 (0.52, 1.45)
3-4 times	0.74 (0.28, 1.19)	1.06 (0.48, 1.63)	1.58 (0.73, 2.44)
5+ times	0.89 (0.30, 1.48)	**0.54 (0.25, 0.83)**	0.82 (0.31, 1.34)
Wave 3			
1-2 times	**1.50 (1.04, 1.95)**	**2.21 (1.55, 2.87)**	1.25 (0.68, 1.83)
3-4 times	**1.59 (1.06, 2.12)**	**2.06 (1.22, 2.90)**	**2.10 (1.14, 3.06)**
5+ times	1.40 (0.88, 1.93)	**2.76 (1.39, 4.12)**	**2.98 (1.47, 4.49)**
**Tobacco use**			
Wave 1			
1-2 times	**0.52 (0.36, 0.68)**	**0.30 (0.16, 0.44)**	**0.55 (0.30, 0.79)**
3-4 times	**0.57 (0.36, 0.79)**	**0.27 (0.16, 0.37)**	**0.30 (0.15, 0.44)**
5+ times	**0.33 (0.18, 0.47)**	**0.28 (0.15, 0.41)**	**0.31 (0.14, 0.48)**
Wave 2			
1-2 times	**1.49 (1.09, 1.88)**	1.02 (0.66, 1.38)	1.00 (0.64, 1.35)
3-4 times	**1.54 (1.12, 1.95)**	0.78 (0.48, 1.07)	0.92 (0.64, 1.20)
5+ times	1.27 (0.93, 1.60)	0.83 (0.55, 1.11)	0.91 (0.60, 1.22)
Wave 3			
1-2 times	**1.49 (1.09, 1.88)**	1.02 (0.66, 1.38)	1.00 (0.64, 1.35)
3-4 times	**1.54 (1.12, 1.95)**	0.78 (0.48, 1.07)	0.92 (0.64, 1.20)
5+ times	1.27 (0.93, 1.60)	0.83 (0.55, 1.11)	0.91 (0.60, 1.22)

Note. The reference group in all models was no engagement. Bold text indicates 95% confidence intervals do not include 1 ($p<0.05$). Early adolescents had a mean age of 13.39 at Wave 1, 14.27 at Wave 2, and 19.74 at Wave 3. Mid adolescents had a mean age of 15.48 at Wave 1, 16.35 at Wave 2, and 21.81 at Wave 3. Late adolescents had a mean age of 17.30 at Wave 1, 18.14 at Wave 2, and 23.55 at Wave 3.

Table S7. Sensitivity analysis: Concurrent associations between hobby engagement frequency in the last week (none, 1-2 times, 3-4 times, 5+ times) and substance use at each wave from the latent growth models, adjusted for all covariates and estimated separately by age group and gender.

	**Early adolescents**		**Mid adolescents**		**Late adolescents**	
	**Males** (n=1,133)	**Females** (n=1,429)	**Males** (n=1,494)	**Females** (n=1,682)	**Males** (n=900)	**Females** (n=816)
	Odds ratio (95% confidence interval)					
**Binge drinking**						
Wave 1						
1-2 times	0.50 (0.20, 1.28)	0.47 (0.22, 1.00)	0.80 (0.37, 1.69)	0.84 (0.45, 1.57)	0.73 (0.36, 1.46)	0.60 (0.25, 1.44)
3-4 times	**0.26 (0.10, 0.69)**	**0.39 (0.16, 0.96)**	**0.40 (0.17, 0.97)**	**0.48 (0.24, 0.95)**	**0.52 (0.28, 0.96)**	0.87 (0.25, 3.08)
5+ times	0.39 (0.15, 1.00)	**0.18 (0.08, 0.39)**	0.54 (0.24, 1.21)	**0.13 (0.06, 0.28)**	**0.36 (0.19, 0.67)**	**0.27 (0.09, 0.83)**
Wave 2						
1-2 times	0.95 (0.40, 2.26)	1.12 (0.64, 1.94)	1.01 (0.63, 1.62)	0.82 (0.51, 1.31)	0.84 (0.44, 1.61)	0.94 (0.41, 2.14)
3-4 times	0.83 (0.38, 1.82)	0.82 (0.47, 1.44)	0.75 (0.38, 1.48)	**0.54 (0.32, 0.92)**	1.17 (0.63, 2.20)	0.65 (0.25, 1.69)
5+ times	0.53 (0.25, 1.12)	0.74 (0.36, 1.48)	**0.54 (0.32, 0.91)**	0.64 (0.35, 1.18)	**0.49 (0.26, 0.92)**	0.66 (0.21, 2.06)
Wave 3						
1-2 times	1.43 (0.86, 2.39)	1.41 (0.98, 2.05)	**2.50 (1.51, 4.15)**	1.23 (0.90, 1.68)	**2.15 (1.22, 3.77)**	**1.70 (1.03, 2.82)**
3-4 times	1.20 (0.65, 2.22)	1.48 (1.02, 2.14)	**1.98 (1.25, 3.13)**	**1.71 (1.17, 2.50)**	**2.20 (1.22, 3.97)**	1.13 (0.72, 1.77)
5+ times	1.09 (0.66, 1.82)	0.86 (0.51, 1.44)	**1.79 (1.09, 2.93)**	**2.14 (1.25, 3.66)**	**2.93 (1.62, 5.27)**	1.15 (0.63, 2.11)
**Marijuana use**						
Wave 1						
1-2 times	**0.36 (0.14, 0.93)**	**0.33 (0.14, 0.75)**	1.21 (0.49, 2.95)	0.54 (0.27, 1.08)	0.59 (0.27, 1.32)	0.62 (0.19, 2.09)
3-4 times	**0.27 (0.11, 0.64)**	**0.27 (0.10, 0.76)**	0.56 (0.20, 1.59)	**0.50 (0.28, 0.90)**	**0.35 (0.13, 0.91)**	0.46 (0.12, 1.77)
5+ times	**0.35 (0.14, 0.90)**	**0.28 (0.10, 0.76)**	0.60 (0.27, 1.33)	**0.24 (0.11, 0.52)**	0.88 (0.25, 3.07)	0.43 (0.08, 2.28)
Wave 2						
1-2 times	1.37 (0.57, 3.30)	1.49 (0.66, 3.39)	1.70 (0.86, 3.39)	0.68 (0.39, 1.19)	0.86 (0.42, 1.73)	0.58 (0.20, 1.67)
3-4 times	0.88 (0.35, 2.20)	0.71 (0.30, 1.64)	**2.20 (1.05, 4.62)**	**0.46 (0.24, 0.89)**	1.27 (0.51, 3.18)	1.45 (0.37, 5.63)
5+ times	1.13 (0.49, 2.59)	0.83 (0.35, 1.93)	0.69 (0.34, 1.41)	**0.41 (0.20, 0.83)**	0.69 (0.27, 1.75)	0.60 (0.11, 3.38)
Wave 3						
1-2 times	1.00 (0.47, 2.13)	1.94 (1.29, 2.92)	1.74 (0.97, 3.12)	**2.16 (1.50, 3.10)**	1.26 (0.62, 2.57)	1.64 (0.66, 4.09)
3-4 times	1.28 (0.58, 2.84)	1.70 (1.04, 2.77)	1.37 (0.72, 2.60)	**2.38 (1.33, 4.25)**	3.53 (2.06, 6.02)	0.99 (0.36, 2.73)
5+ times	1.29 (0.62, 2.73)	1.26 (0.71, 2.23)	**2.54 (1.39, 4.64)**	**2.37 (1.12, 5.00)**	**3.45 (1.66, 7.16)**	3.09 (0.97, 9.83)
**Tobacco use**						
Wave 1						
1-2 times	**0.43 (0.23, 0.80)**	**0.62 (0.43, 0.90)**	**0.46 (0.25, 0.87)**	**0.21 (0.13, 0.34)**	0.52 (0.22, 1.21)	**0.31 (0.15, 0.66)**
3-4 times	**0.36 (0.21, 0.64)**	0.95 (0.55, 1.65)	**0.43 (0.22, 0.84)**	**0.16 (0.10, 0.26)**	0.62 (0.29, 1.35)	**0.10 (0.04, 0.24)**
5+ times	**0.34 (0.19, 0.60)**	**0.31 (0.18, 0.54)**	**0.42 (0.22, 0.80)**	**0.18 (0.11, 0.31)**	**0.23 (0.11, 0.49)**	0.57 (0.21, 1.58)
Wave 2						
1-2 times	1.08 (0.69, 1.70)	**2.04 (1.39, 2.99)**	0.97 (0.66, 1.45)	1.00 (0.70, 1.41)	0.84 (0.52, 1.36)	1.31 (0.66, 2.59)
3-4 times	1.22 (0.74, 2.00)	**1.88 (1.29, 2.75)**	0.92 (0.65, 1.32)	**0.56 (0.34, 0.92)**	1.01 (0.63, 1.63)	0.94 (0.46, 1.90)
5+ times	0.93 (0.61, 1.42)	**1.83 (1.26, 2.68)**	1.00 (0.69, 1.44)	**0.60 (0.39, 0.93)**	0.79 (0.48, 1.30)	1.26 (0.58, 2.70)
Wave 3						
1-2 times	1.08 (0.69, 1.70)	**2.04 (1.39, 2.99)**	0.97 (0.66, 1.45)	1.00 (0.70, 1.41)	0.84 (0.52, 1.36)	1.31 (0.66, 2.59)
3-4 times	1.22 (0.74, 2.00)	**1.88 (1.29, 2.75)**	0.92 (0.65, 1.32)	**0.56 (0.34, 0.92)**	1.01 (0.63, 1.63)	0.94 (0.46, 1.90)
5+ times	0.93 (0.61, 1.42)	**1.83 (1.26, 2.68)**	1.00 (0.69, 1.44)	**0.60 (0.39, 0.93)**	0.79 (0.48, 1.30)	1.26 (0.58, 2.70)

Note. The reference group in all models was no engagement. Bold text indicates 95% confidence intervals do not include 1 (p<0.05). Early adolescents had a mean age of 13.39 at Wave 1, 14.27 at Wave 2, and 19.74 at Wave 3. Mid adolescents had a mean age of 15.48 at Wave 1, 16.35 at Wave 2, and 21.81 at Wave 3. Late adolescents had a mean age of 17.30 at Wave 1, 18.14 at Wave 2, and 23.55 at Wave 3.

Table S8. Sensitivity analysis: Evaluating the implications of altering the sample eligibility criteria and approach to missing data on the concurrent associations between hobby engagement frequency and binge drinking at each wave from the latent growth models, adjusted for all covariates, estimated just for early adolescents.

	**Original** (n=2,562)	**Sensitivity 1** (n=4,961)	**Sensitivity 2** (n=3,471)	**Sensitivity 3** (n=2,413)
	Odds ratio (95% confidence interval)			
**Early adolescents: Binge drinking**				
Wave 1				
1-2 times	**0.47 (0.21, 0.73)**	**0.46 (0.31, 0.69)**	**0.44 (0.29, 0.66)**	0.44 (0.19, 1.03)
3-4 times	**0.32 (0.12, 0.52)**	**0.36 (0.23, 0.56)**	**0.33 (0.21, 0.53)**	0.43 (0.11, 1.74)
5+ times	**0.29 (0.12, 0.46)**	**0.33 (0.23, 0.47)**	**0.30 (0.19, 0.46)**	**0.30 (0.13, 0.68)**
Wave 2				
1-2 times	1.04 (0.58, 1.50)	0.98 (0.66, 1.46)	1.01 (0.69, 1.47)	1.14 (0.29, 4.44)
3-4 times	0.81 (0.43, 1.20)	0.77 (0.52, 1.13)	0.76 (0.51, 1.15)	0.90 (0.13, 6.17)
5+ times	**0.60 (0.28, 0.91)**	0.69 (0.45, 1.04)	**0.66 (0.41, 1.06)**	0.81 (0.12, 5.37**)**
Wave 3				
1-2 times	1.41 (0.97, 1.85)	**1.38 (1.09, 1.76)**	**1.37 (1.06, 1.77)**	**1.38 (1.03, 1.85)**
3-4 times	1.34 (0.84, 1.83)	1.38 (0.99, 1.91)	1.35 (0.96, 1.91)	1.44 (0.87, 2.38)
5+ times	0.98 (0.62, 1.35)	0.99 (0.72, 1.37)	0.98 (0.71, 1.35)	1.25 (0.71, 2.20)

Note. Original: Sample restricted to participants with no missing data on any study variables at waves 1-3, but missing data on substance use at waves 4-5 handled using FIML. Early adolescent group only (n=2,562).
Sensitivity 1: Sample included all participants who participated in at least one wave and reported their age at baseline, with all missing data handled using FIML. Early adolescent group only (n=4,961).
Sensitivity 2: Sample restricted to participants who participated at waves 1-3 with complete baseline age data, with all missing data handled using FIML. Early adolescent group only (n=3,471).
Sensitivity 3: Sample restricted to participants who participated at waves 1-5 with complete baseline age data, with all missing data handled using FIML. Early adolescent group only (n=2,413).

Mplus Model Syntax

Final model: Binge drinking

```
Data:
  File = ah_subuse.dat ;

Variable:
  Names =
    aid psuscid region gswgt3 insamp w1hobby1 w1hobby2 w1hobby3 w1hobby4
    w2hobby1 w2hobby2 w2hobby3 w2hobby4 w3hobby1 w3hobby2 w3hobby3 w3hobby4
    age gender grade hinc lang psmoke palc race1 race2 race3 race4
    pedu1 pedu2 pedu3 pedu4 pmarit1 pmarit2 pmarit3 urban1 urban2 urban3
    w1drink w2drink w3drink w4drink w5drink w1mar w2mar w3mar w4mar w5mar
    w1tob w2tob w3tob w4tob w5tob ;

  Missing =
    all (-999) ;

  Usevar =
    w1drink w2drink w3drink w4drink w5drink
    w1hobby2 w1hobby3 w1hobby4 w2hobby2
    w2hobby3 w2hobby4 w3hobby2 w3hobby3 w3hobby4
    age gender grade hinc lang psmoke palc race2 race3 race4
    pedu1 pedu2 pedu3 pmarit2 pmarit3 urban2 urban3
    gswgt3 psuscid region ;

  Categorical =
    w1drink w2drink w3drink w4drink w5drink ;

  Weight =
    gswgt3 ;

  Cluster =
    psuscid ;

  Strat =
    region ;

  Subpopulation =
     insamp == 1 ;

Analysis:
  Estimator = MLR ;

  Type = complex ;

Model:
  i sl |      w1drink@0 w2drink@1 w3drink* w4drink* w5drink* ;

  i sl ON     age gender grade hinc lang psmoke palc
              race2 race3 race4 pedu1 pedu2 pedu3 pmarit2 pmarit3 urban2 urban3 ;

  w1drink ON  w1hobby2 w1hobby3 w1hobby4 ;

  w2drink ON  w2hobby2 w2hobby3 w2hobby4 ;

  w3drink ON  w3hobby2 w3hobby3 w3hobby4 ;

Output:
  TECH1 ;
  TECH4 ;
  TECH8 ;
  RESIDUAL ;
```

Final model: Marijuana use

```
Data:
  File = ah_subuse.dat ;

Variable:
  Names =
    aid psuscid region gswgt3 insamp w1hobby1 w1hobby2 w1hobby3 w1hobby4
    w2hobby1 w2hobby2 w2hobby3 w2hobby4 w3hobby1 w3hobby2 w3hobby3 w3hobby4
    age gender grade hinc lang psmoke palc race1 race2 race3 race4
    pedu1 pedu2 pedu3 pedu4 pmarit1 pmarit2 pmarit3 urban1 urban2 urban3
    w1drink w2drink w3drink w4drink w5drink w1mar w2mar w3mar w4mar w5mar
    w1tob w2tob w3tob w4tob w5tob ;

  Missing =
    all (-999) ;

  Usevar =
    w1mar w2mar w3mar w4mar w5mar
    w1hobby2 w1hobby3 w1hobby4 w2hobby2
    w2hobby3 w2hobby4 w3hobby2 w3hobby3 w3hobby4
    age gender grade hinc lang psmoke palc race2 race3 race4
    pedu1 pedu2 pedu3 pmarit2 pmarit3 urban2 urban3
    gswgt3 psuscid region ;

  Categorical =
    w1mar w2mar w3mar w4mar w5mar ;

  Weight =
    gswgt3 ;

  Cluster =
    psuscid ;

  Strat =
    region ;

  Subpopulation =
     insamp == 1 ;

Analysis:
  Estimator = MLR ;

  Type = complex ;

Model:
  i sl |      w1mar@0 w2mar@1 w3mar* w4mar* w5mar* ;

  i sl ON     age gender grade hinc lang psmoke palc
             race2 race3 race4 pedu1 pedu2 pedu3 pmarit2 pmarit3 urban2 urban3 ;

  w1mar ON    w1hobby2 w1hobby3 w1hobby4 ;

  w2mar ON    w2hobby2 w2hobby3 w2hobby4 ;

  w3mar ON    w3hobby2 w3hobby3 w3hobby4 ;

Output:
  TECH1 ;
  TECH4 ;
  TECH8 ;
  RESIDUAL ;
```

Final model: Tobacco use

```
Data:
  File = ah_subuse.dat ;

Variable:
  Names =
    aid psuscid region gswgt3 insamp w1hobby1 w1hobby2 w1hobby3 w1hobby4
    w2hobby1 w2hobby2 w2hobby3 w2hobby4 w3hobby1 w3hobby2 w3hobby3 w3hobby4
    age gender grade hinc lang psmoke palc race1 race2 race3 race4
    pedu1 pedu2 pedu3 pedu4 pmarit1 pmarit2 pmarit3 urban1 urban2 urban3
    w1drink w2drink w3drink w4drink w5drink w1mar w2mar w3mar w4mar w5mar
    w1tob w2tob w3tob w4tob w5tob ;

  Missing =
    all (-999) ;

  Usevar =
    w1tob w2tob w3tob w4tob w5tob
    w1hobby2 w1hobby3 w1hobby4 w2hobby2
    w2hobby3 w2hobby4 w3hobby2 w3hobby3 w3hobby4
    age gender grade hinc lang psmoke palc race2 race3 race4
    pedu1 pedu2 pedu3 pmarit2 pmarit3 urban2 urban3
    gswgt3 psuscid region ;

  Categorical =
    w1tob w2tob w3tob w4tob w5tob ;

  Weight =
    gswgt3 ;

  Cluster =
    psuscid ;

  Strat =
    region ;

  Subpopulation =
     insamp == 1 ;

Analysis:
  Estimator = MLR ;

  Type = complex ;

Model:
  i sl |      w1tob@0 w2tob@1 w3tob* w4tob* w5tob* ;

  i sl ON     age gender grade hinc lang psmoke palc
              race2 race3 race4 pedu1 pedu2 pedu3 pmarit2 pmarit3 urban2 urban3 ;

  w1mar ON    w1hobby2 w1hobby3 w1hobby4 ;

  w2mar ON    w2hobby2 w2hobby3 w2hobby4 ;

  w3mar ON    w3hobby2 w3hobby3 w3hobby4 ;

Output:
  TECH1 ;
  TECH4 ;
  TECH8 ;
  RESIDUAL ;
```
